# Smart approach for international normalized ratio monitoring: Pakistan’s perspective

**DOI:** 10.1097/MS9.0000000000002419

**Published:** 2024-08-01

**Authors:** Usha Kumari, Shanzay Zahid, Aarash Khan

**Affiliations:** a Dow University of Health Sciences, Karachi, Pakistan; b Herat University, Afghanistan

The advent of direct-acting oral anticoagulants (DOACs) has largely replaced warfarin, a vitamin K antagonist, as a therapy of choice for the prevention and management of thrombosis in many clinical indications. However, warfarin is still commonly prescribed due to financial restraints and contraindications associated with DOACs^1^. Management of thrombotic diseases with warfarin requires close monitoring of the international nationalized ratio (INR) due to its narrow therapeutic index, complex pharmacokinetics, and frequent drug interactions^2,3^. Periodic monitoring of INR level has proven to reduce many complications such as stroke, bleeding, or thromboembolic events while at the same time undergoing these periodic checks via traditional laboratory-based methods may entail discomfort for the patients^3–5^.

Several studies have found that a lack of understanding of anticoagulation therapy among patients is associated with poor clinical outcomes^6^. A cross-sectional study from Pakistan highlights that the level of awareness about regular INR monitoring and anticoagulation therapy is highly substandard in Pakistan, with about 78.9% of the study population being unaware of their target INR range^7^. A similar gap in knowledge has been reported in a study conducted in Nepal^8^ and Hong Kong^6^ and is attributed to the lack of patient education and regular counseling^7^. Shabneez *et al*.^9^ observed communication failure to be responsible for a delay in notifying critical INR values to the patients and subsequently increased turnaround time for results.

The introduction of point-of-care testing (POCT) with portable devices like CoaguCheck (CoC), along with the establishment of thrombosis centers can significantly aid in the monitoring of anticoagulation therapy^5^. It is analyzed that 92% of patients who monitor their INR levels every 3 days with CoC successfully remain in their therapeutic range compared to 50–60% of patients who monitor their INR levels through conventional lab technique^10^. Likewise, a study conducted in remote and rural regions of Australia demonstrated the effectiveness of point-of-care INR testing, as levels of monitoring and surveillance were adequate and warfarin medication complications were low^11^.

The CoC device offers a reliable alternative to laboratory testing of INR. This innovative device measures the prothrombin time and INR using a small capillary blood sample obtained via finger prick^2^. Consisting of a compact coagulometer and disposable test strips, it utilizes electrochemical energy to draw blood into a microchannel and detects clot formation, providing INR results within a few minutes^2^. Recent advancements include Bluetooth technology integration, facilitating the secure and accurate transfer of INR results to hospital databases. This reduces overall turnaround time for INR monitoring, improving the management of patients undergoing long-term warfarin therapy^5,12,13^.

Comparative studies have demonstrated a strong correlation between POCT and conventional laboratory techniques for measuring INR^14^. Cost analyses suggest that integrating CoC devices into healthcare systems is a cost-effective strategy for home INR testing^15^. This integration has also demonstrated significant clinical benefits, with reported reductions in thromboembolic events by 42–55% and all-cause mortality by 26–42%^16^. Moreover, the establishment of anticoagulation call centers (ACCs) can further enhance patient care by enabling remote access services for communicating POCT results with healthcare professionals directly from patients’ homes. ACCs facilitate swift treatment initiation, dosage adjustment, or discontinuation, ensuring regular follow-ups and providing vital patient counseling, especially for individuals facing challenges in accessing hospital facilities.

Patient self-testing and self-management have shown great improvement in patients’ and their families’ quality of life^17^. However, there are certain limitations surrounding the implementation of home-based POCT, including connectivity issues, technology issues, reliability of the device, necessary training requirements, and coordination of data collection^18^. However, in the wake of the rising healthcare demand, POCT systems present a greater societal economic benefit as compared to traditional models and are an effective strategy for INR monitoring^17,18^.

## Ethical approval

Ethics approval was not required for this editorial.

## Consent

Informed consent was not required for this editorial.

## Source of funding

No source of funding to declare.

## Author contribution

U.K.: idea, design, and draft writing; S.Z.: writing and editing; A.K.: project administration and reviewing.

## Conflicts of interest disclosure

The authors declare no conflicts of interest.

## Research registration unique identifying number (UIN)

Not applicable.

## Guarantor

Usha Kumari.

## Data availability statement

Not applicable.

## Provenance and peer review

Not commissioned, externally peer-reviewed.
